# Biosignature of self-injury behaviors in adolescence: Role of β-endorphin in an acute inpatient unit

**DOI:** 10.3389/fpsyt.2022.933275

**Published:** 2022-08-15

**Authors:** Ping Wang, Chao Li, Pablo del Sol-Calderón, Leticia Mallol, Elena Hernández-Álvarez, Encarnación Donoso-Navarro, María Gil-Ligero, Silvia Rosado-Garcia, Antonio José Sánchez-Lòpez, Marina Martín-Moratinos, Marcos Bella-Fernández, Hilario Blasco-Fontecilla

**Affiliations:** ^1^Faculty of Medicine, Autonomous University of Madrid, Madrid, Spain; ^2^Department of Psychiatry, Puerta de Hierro University Hospital, Health Research Institute Puerta de Hierro-Segovia de Arana (IDIPHISA), Madrid, Spain; ^3^Department of Clinical Biochemistry, Puerta de Hierro University Hospital, IDIPHISA, Madrid, Spain; ^4^Biobank, Puerta de Hierro-Segovia de Arana Health Research Institute, Madrid, Spain; ^5^Neuroimmunology Unit, Puerta de Hierro-Segovia de Arana Health Research Institute, Madrid, Spain; ^6^Department of Psychology, Pontifical University of Comillas, Madrid, Spain; ^7^Center of Biomedical Network Research on Mental Health (CIBERSAM), Madrid, Spain; ^8^Korian, Paris, France

**Keywords:** self-injury behavior, non-suicidal self-injury, suicidal behavior, addiction, β-endorphin, adolescents

## Abstract

Self-injurious behavior (SIB) (either non-suicidal self-injury, NSSI; or suicide attempts, SA) is a common reason for adolescent psychiatric emergency hospitalizations. Altered basal serum β-endorphin (BE) levels have been reported in adults with a history of SIB, but information is lacking in adolescents. We analyzed the psychoclinical profile and serum BE level of 39 adolescents admitted to the acute unit at a hospital in Spain due to SIB. The Mean (SD) serum BE level was high (190.53 ± 74.83). Regarding time sequence, the onset age of NSSI and SA were related (*p* < 0.001). The older the onset age of NSSI, the shorter the transition between NSSI and the onset of SA behavior (*p* = 0.05), but this difference does not lead the variation of BE (*p* = 0.81). Patients diagnosed with depression had lower serum BE levels than adolescents with other diagnoses (*p* = 0.03). Although adolescents who seem to be addicted to SIB had higher levels of BE, this finding was not statistically significant. The relationship between serum BE levels and SIB in adolescents requires further investigation.

## 1. Introduction

Self-injurious behavior (SIB) includes a wide range of behaviors, including some degree of physical or psychological self-damage. SIB includes non-suicidal self-injury (NSSI) and suicidal behavior (suicidal ideation, suicide plan, and suicide attempts) (1, 2). SIB is highly prevalent among adolescents and young adults. It is estimated that between 17.1% and 38.6% of adolescents experience lifetime SIB, and 7.83% repetitively (3). Individuals who engage in SIB are more likely to have mental disorders (4, 5). Noteworthy, SIB increases family health risks and the need for social services and medical attention (6). Although recent studies have yielded important insights into the prevalence, etiology, evaluation, and treatment of SIB (7, 8), the prediction and prevention of SIB remain challenging. A critical task in preventing SIB is to identify markers of risk for subsequent SIB to target prevention. Accordingly, the study of the role of biomarkers in SIB adolescents may have the potential value of identifying a high-risk group and helping to prevent suicide (9).

SIB leads to physical harm and pain; however, paradoxically, most self-injurers report feeling lower pain intensity (10, 11). Therefore, some researchers have suggested that altered pain processing may be a potential risk factor for SIB (2). Serum β-endorphin (BE) is thought to play an important role in SIB as BE regulates injury perception and analgesia in the body. The opioid deficiency hypothesis (12–14) argues that people who engage in SIB have low basal BE levels and raise them through repeated SIB. The addiction hypothesis proposes that people who exhibit SIB may be addicted to their own endogenous opioids (15). BE is known to induce feelings of euphoria and happiness by modulating dopamine neurons in parts of the brain associated with motivation and pleasure and influencing positive emotions to reward and reinforce behavior (16). SIB is thought to be an extreme response resulting from regulatory control in the face of negative emotions or stressful events (17). Thus, the release of BE activated by self-injury may help to reduce feelings of pain and increase pleasure (12, 18). This may lead to the addiction to SIB in vulnerable subjects (19). Indeed, our group has reported that adults addicted to suicidal behavior displayed higher serum BE levels than adult patients who are not addicted to suicidal behavior (20).

Previous studies have shown altered basal BE levels in adults with a history of NSSI (21). Unfortunately, very few studies targeted BE levels in adolescent self-injurers. This population is characterized by repetitive SIB, comorbidity, and a high risk of suicide (7). This study examines the relationship between psychopathology and BE levels with SIB. We hypothesized that: 1. Mental disorders mediate the relationship between BE levels and SIB; 2. Both the SIB and criteria of addiction to SIB correlate with BE levels, consistent with the addictive theory of suicide addiction.

## 2. Methods

### 2.1. Sample and procedure

Sixty-two acute inpatient adolescents at Puerta de Hierro University Hospital (Majadahonda, Madrid, Spain) with SIB were enrolled between February 25, 2021, and February 23, 2022. Inclusion criteria were as follows: inpatients with SIB; age 12–17 years, and competent in Spanish. Exclusion criteria were as follows: patients with any disease-causing endocrine disorders, such as diabetes mellitus, hypothyroidism, or being treated with hormones (except oral contraceptives); no parent/guardian to consent or no valid values of BE. All participants and their legal guardians signed an informed consent form after the explanation of the study. The local ethics committee approved the study (IRB Number 82/20, 23rd February 2021). A protocol was designed by the principal investigator for data collection, who also evaluated all participants to collect sociodemographic and clinical data. The Electronic Medical Records were reviewed to complete the information included in the protocol. Biological assessments and clinical interviews were performed within 24–48 h of patient admission. Eventually, 39 patients who met inclusion criteria in whom BE levels were available were included in the analyses.

### 2.2. Measures

Apart from the serum BE level, the main measurements included the clinical diagnosis of patients, characteristics and risk factors of SIB. The primary diagnosis of the patients were a clinical diagnosis based on DSM-5 criteria.

The number and age characteristics of SIB included the onset age of NSSI and SA as well as the number of NSSI and SA. The Paykel Suicide Scale (PSS) (22) and four items of the Self-Injurious Thoughts and Behaviors Inventory (SITBI) (23) were used to evaluate some suicidal characteristics. In addition, the study also used the Short Personality and Life Event Scale (S-PLE), a 6-item instrument, which demonstrated good accuracy and sensitivity to suicide attempters in adult samples (24), as well as a criterion for SIB addiction modified from DSM-5 criteria for substance addiction. This criterion contains 11 items divided into four sections (control of disturbing behavior, social difficulty, risk use, and pharmaceutical criteria) (25). Lastly, the Unbearable Psychache Scale (UP3), a 3-item scale (score range 0–15) was used to assess unbearable psychache (26).

#### 2.2.1. Blood collection

Blood samples were collected between 7:30 and 8:30 a.m. on the day after admission at the acute inpatient unit. The serum BE (pg/mL) was evaluated using a commercially available enzyme-linked immunosorbent assay (ELISA) Kit [Cloud-Clone Corp (CEA806Hu), range 12.35–1,000 pg/ml] according to the manufacturer's instructions. BE levels were measured in serum. Serum samples collected were anonymized to keep the laboratory staff blind to clinical variables. Samples and data from patients included in this study were provided by the Majadahonda Puerta de Hierro University Hospital Biobank (HUPH)/Segovia de Arana Puerta de Hierro University Hospital Research Institute (IDIPHISA) integrated within the National Spanish Biobank Network. They were processed following standard operating procedures with the appropriate approval of the Ethics and Scientific Committees.

### 2.3. Statistical analysis

After applying the inclusion and exclusion criteria, 39 patients were included for analysis. No patient had missing values in more than 20% of all variables. The main analysis focused on the relationship between BE and other measures. Pearson's r or Spearman's rank correlation coefficient was used to describe the correlations between continuous variables. In case of necessity, some continuous variables could be transformed into categorical variables according to certain criteria to discuss their relationship with BE. Categorical variables were analyzed using Analysis of Variance (ANOVA) or Kruskal–Wallis ANOVA. Analysis of covariance (ANCOVA) or linear models was also used when necessary. R version 4.1 (27) was used for the analysis.

## 3. Results

Most included patients were born in Spain (*n* = 34), and the majority were Caucasians (*n* = 31). Eighty-seven percent (*n* = 34) were girls. The mean age of the sample was 15 years (14.92 ± 1.49). Fifteen percent (*n* = 6) of these adolescents were the only children in their families (see Table 1). The mean serum BE was 190.53 ± 74.83. Age positively correlated with BE levels (*p* = 0.03). The most frequent clinical diagnosis was depression (*n* = 25), followed by ADHD (*n* = 7). BE levels were lower in depressed patients (*F* = 3.96, df = 2, *p* = 0.03) and higher in patients diagnosed with ADHD and other diagnoses (see **Figure 2A**).

**Table 1:** Main features of the sample (*n* = 39).

**Variables**	**Mean ±SD or *N* (%)**	**Statistic^***a***^**	***P*-value**
**Gender**		*F* = 0.35	0.56
Female	34 (87)		
Male	5 (17)		
**Birthplace**		*F* = 0.32	0.57
Spain	34 (87)		
Others	5 (13)		
**Ethnic**		*F* = 0.41	0.53
Caucasian	31 (79)		
Others	8 (21)		
**Family type**		*F* = 1.65	0.20
One-child family	6 (15)		
Multi-child family	33 (85)		
**Age (yr)**	14.92 ± 1.49	RS = 0.34	0.03
**Age of first NSSI**	12.97 ± 1.55	RP = −0.03	0.87
**Age of first suicidal attempt (*****n*** **=** **32)**	13.59 ± 1.66	RS = 0.10	0.57
**Transition Modes** ^***b***^		*F* = 0.06	0.81
Quick transition	14 (36)		
Slow transition	23 (59)		
Unknown	2(5)		
**Primary diagnosis (*****n*** **=** **38)**		
Depressive episode	25 (66)	*F* = 8.13	<0.01
ADHD	7 (18)	*F* = 3.30	0.08
Others	6(16)	/	/
**PSS**^***c***^ **(*****n*** **=** **38)**	4.76 ± 0.43	RS = −0.05	0.78
**SITBI**^***d***^ **(*****n*** **=** **38)**	10.21 ± 2.60	RP = −0.05	0.77
**S-PLE** ^***e***^	2.97 ± 0.36	RS = −0.42	<0.01
**UP3**^***f***^ **(*****n*** **=** **38)**	12.68 ± 2.52	RS = 0.09	0.61
**DSM-5 criteria for addiction**^***g***^ **(*****n*** **=** **38)**	7.89 ± 2.41	RS = −0.05	0.78
Control of disturbed behavior	3.21 ± 0.99	RS = −0.19	0.25
Social difficulty	1.89 ± 0.98	RS = 0.05	0.78
Use in risk	1.37 ± 0.71	RS = 0.04	0.83
Pharmacological criteria	1.42 ± 0.64	RS = −0.07	0.70
**Beta-endorphin (serum)**	190.53 ± 74.83	/	/

a RS, Spearman's rank correlation coefficient; RP, Pearson's correlation coefficient; F, F statistic of ANOVA.

The correlation between variable and beta-endorphin level was shown in the table.

b In the transition mode, the “Slow Transition” is considered if the patient requires at least 1 year to transition from NSSI to SA behavior (including cases where the patient still does not have SA but more than a year has passed since he/she had NSSI); The “Quick Transition” is considered if the patient had SA before NSSI or both behaviors appeared at the same age. The “Unknown” group consisted of patients with their first NSSI but no SA in the recent year.

c PSS, The Paykel Suicide Scale. All possess a minimum of four points on the PSS scale. ANOVA was used to assess the difference in beta-endorphin levels between those with a score of 4 and those with a score of 5.

d SITBI: Included questions 41–44 of the self-injurious thoughts and behaviors inventory scale.

e S-PLE, the short personality and life event scale.

f UP3, the unbearable psychache scale.

g The criteria are modified by DSM-5 criteria for substance addiction.

**Figure 1:**
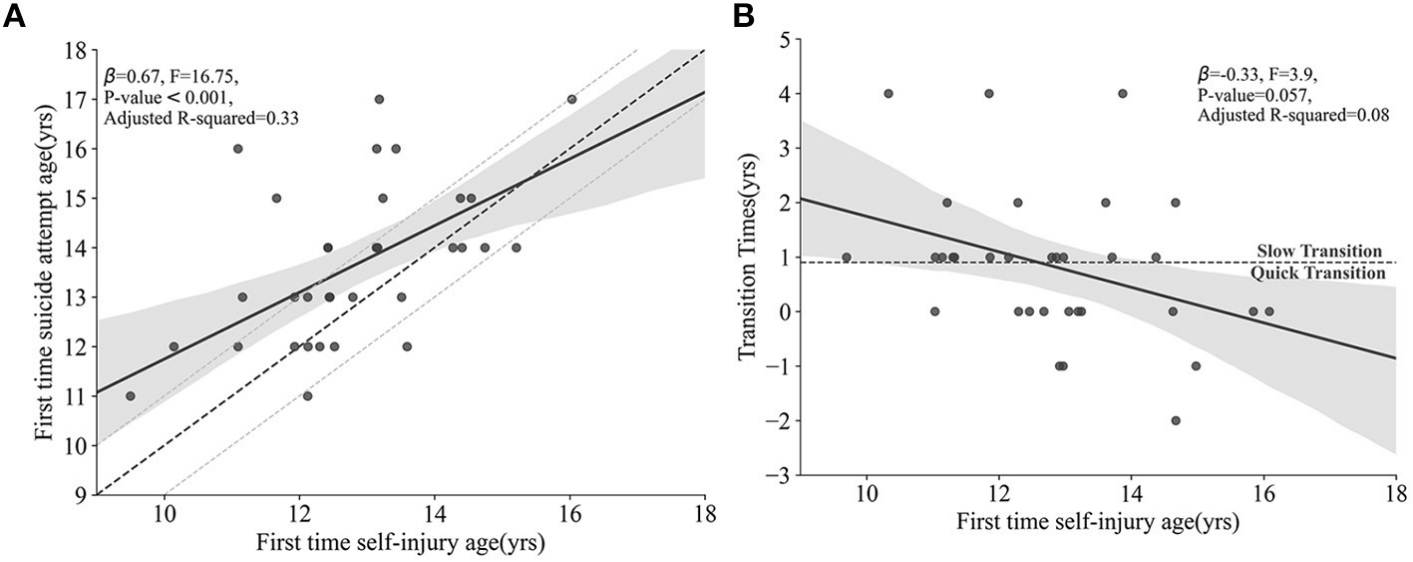
**(A)** The association between the onset age of NSSI and SA. The solid black line slowly approaches the diagonal dashed line in the figure, which is the line that fits the appearing NSSI and SA at the same age. **(B)** The association between the transition time from NSSI to SA and the onset age of NSSI. The area below the dashed line indicates the “Quick Transition” mode (time required from the onset of NSSI to SA was <1 year); the area above the dashed line indicates “Slow Transition” mode (time required from the onset of NSSI to SA is ≥1 year). NSSI, non-suicidal self-injury; SA, suicide attempt.

The mean onset age of NSSI was 13 years old (12.97 ± 1.55), Eighty-three percent (*n* = 33) of the patients also experienced SA, and the mean onset age of SA was 13 years old (13.67 ± 1.61). Seventy-four percent (*n* = 29) picked all PSS items with risky responses. No correlation between the PSS and SITBI with BE levels was detected. There was an association between the onset age of NSSI and the onset age of SA (*p* = 0.001, see Figure 1A), and the older the onset age of NSSI, the shorter the period between NSSI and the onset of SA (see Figure 1B). Based on this feature, we propose a classification: (1) the “Quick Transition” group: patients who began SA within 1 year after their first NSSI episode or who began their SIB career with a suicide attempt; and (2) the “Slow Transition” group: patients who passed more than 1 year between their first NSSI episode and their first SA. In addition, the “Unknown” group was comprised of patients who had begun NSSI <1 year before our data collection and had no SA. No difference was found between the quick transition group and the slow transition group (194.42 ± 83.77 vs. 187.96 ± 69.23, *p* = 0.81).

**Figure 2:**
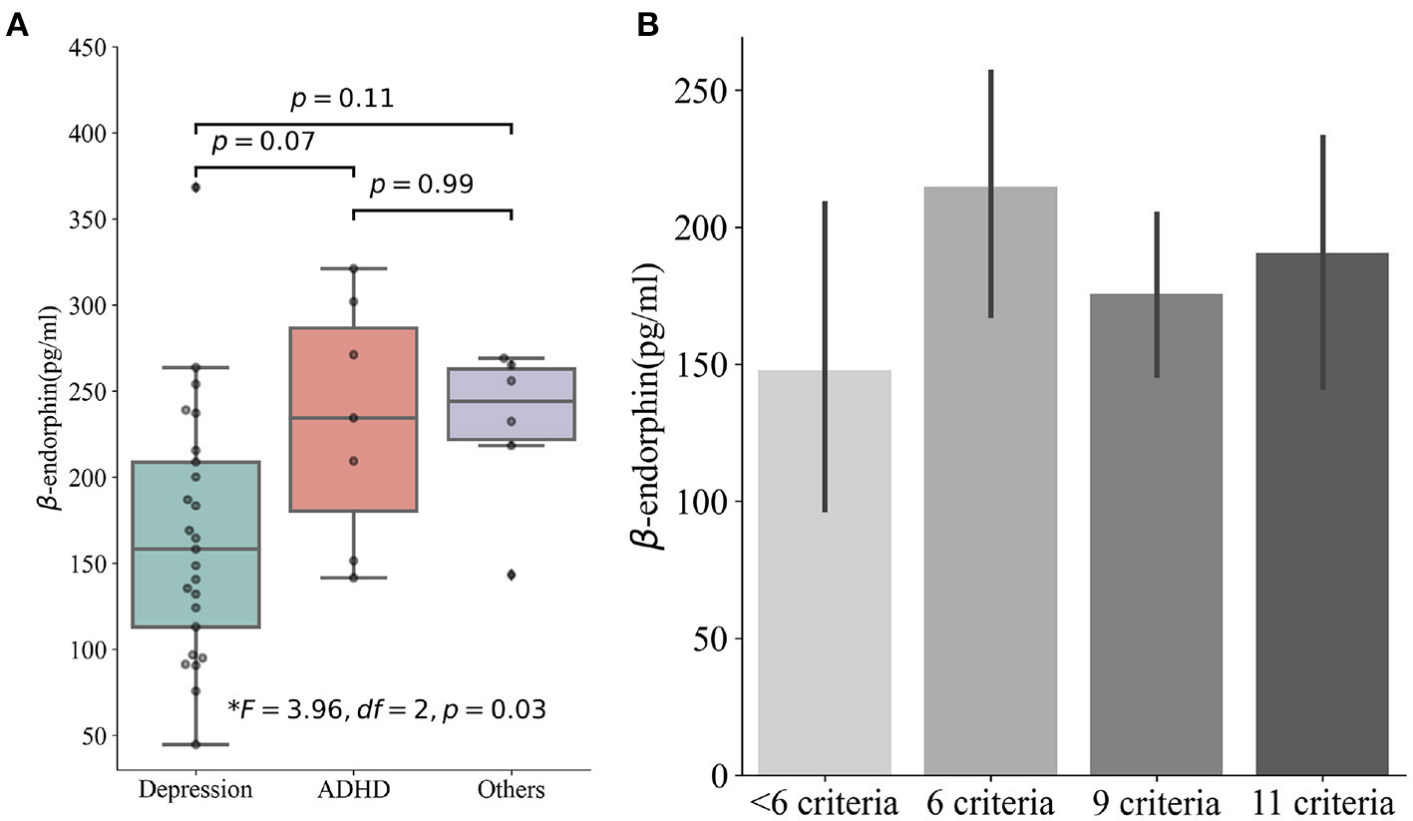
**(A)** Patients with different clinic diagnoses and their differences in serum beta-endorphins. **(B)** Patients met different SIB addiction criteria and their differences in serum beta-endorphins. SIB, self-injurious behavior.

We also found a negative correlation between the S-PLE scores and BE levels in a simple linear regression model (β = −86.78, *F* = 7.98, *p* = 0.008, Adj. *R*^2^ = 0.16). Half (*n* = 19) of the adolescents in the sample chose all answers that increased the risk of SA, except the question “*If you have a partner, do you think he/she is unfaithful?”*. In the DSM-IV-based addictive SIBs scale, eighty-seven percent (n = 33) of the participants fulfilled at least 6 out of the 11 criteria of SIB addiction; however, we found no statistically significant differences when comparing those addicted to SIBs to those not addicted (196.17 ± 74.19 vs. 147.73 ± 63.48, *F* = 1.82 *p* = 0.19; see Figure 2B). This difference may be statistically significant if the sample size increased to 123 individuals (assuming *p* = 0.05 and power = 0.8).

## 4. Discussion

The present study reports several interesting findings regarding the association of serum BE values and sociodemographic and clinic characteristics in a sample of 39 adolescents hospitalized for SIB. The most relevant clinical finding was that the transition time from NSSI to SA is shorter with increasing age of onset at first NSSI. Regarding serum BE values, we found several interesting findings. First, the serum BE values were surprisingly high. Second, adolescents diagnosed with depression had lower serum BE values than those with other diagnoses, particularly ADHD. Third, the S-PLE global score correlated with serum BE values. Nevertheless, another interesting result even if it did not reach statistical significance, was that serum BE values increased with a higher degree of addiction to SIB.

Previous studies have reported that NSSI is an important factor for subsequent suicide behaviors (28, 29). In the present study, most patients who presented with NSSI also had at least one SA; a few patients had SA earlier than NSSI. Regarding the time sequence, the onset age of NSSI and SA were related. The earlier onset of NSSI is more likely to take a longer NSSI-SA transition time. Some studies suggested that this difference may be because those with earlier onset of NSSI lack the measures and knowledge to carry out SA earlier (30, 31). The BE did not differ between those who require longer NSSI-SA transition time (“Slow Transition”) and those who require less time (“Quick Transition”) to begin carrying out SA. Although more study is required, it seems that the transition between NSSI and SA does not lead to the variation of BE.

We found reduced BE levels in depressed patients, which is consistent with the findings of several previous studies (11, 32, 33). Nonetheless, some studies also reported opposite results (34, 35). One possible explanation is that the methodologies, sample gender and age, treatment medications, and screening for depression comorbidity possible conduce to different outcomes (36). Some studies showed that BE decreased in ADHD patients (37, 38). Our patients with ADHD as their primary diagnosis did not show the same decrease compared to other disorders. This may be because most ADHD patients in our sample were treated with medication, or because some medications could raise serum BE values (39).

In the analysis of other characteristics associated with SIB, the BE level significantly decreased as the S-PLE score increased. For adults, a score higher than 2.46 (1.70 has also been recommended as a cut-off point for increasing sensitivity) was interpreted as a high risk of suicidal attempt (40, 41). However, using S-PLE in the underage population still lacks study. Some questions are inappropriate for the adolescent population, for example, the question “If you had a partner, do you think he/she was unfaithful?” may sounds awkward for majority of adolescents. Therefore, the inverse relationship between S-PLE and BE serum levels should be interpreted cautiously. Finally, adolescents who met at least six criteria for SIB addiction had greater BE levels than those who met fewer criteria. However, for significant results, the expected sample should be >100, and patients in our study were mostly depressed. The effect of depression could overlap the difference in BE by different levels of addiction. Moreover, most adolescents in our sample had SIB for <3 years and the episodes were infrequent. A longitudinal study with a larger sample size may lead to significant results. In any case, a previous study of our team demonstrated a relationship between meeting criteria for an addiction to suicidal behavior and BE values.

This study aimed to discover potential variables related to variations in serum BE levels in a sample of adolescents hospitalized with SIB. Analysis of the characteristics of SIB and potential biomarkers may provide insight into the prevention of lifetime SIB. Our findings indicate that variations in BE levels are frequently related to multiple factors, although the limitations of the sample size prevented us to carry out some extra analyses that could reveal complex relationships. Future research using a larger sample and a longitudinal design with multiple measures is required to better comprehend the variations and peculiarities of BE serum values in adolescents. Moreover, we used interviews with patients and self-reports to collect questions related to SIB, which may be subject to biases such as concealment, exaggeration, and memory bias. Also, self-reported measures may reflect individual feelings and impressions rather than objective measures. Third, we lacked a control group which resulted in highly homogenized data and limited results. Fourth, our sample is gender-biased in the sense that most participants are female, which in turn makes our conclusions less generalizable and limits the identification of sex differences. However, as our study is still ongoing, a larger, balanced sample may lead to more significant results and allow more sophisticated analyses, such as Structural Equation Modeling analysis and machine learning-based models.

## Data availability statement

The raw data supporting the conclusions of this article will be made available by the authors, without undue reservation.

## Ethics statement

The studies involving human participants were reviewed and approved by Comité de Ética de Investigación y Comité de Etica de Investigación con Medicamentos (CEIm). Written informed consent to participate in this study was provided by the participants' legal guardian/next of kin. Written informed consent was obtained from the individual(s), and minor(s)' legal guardian/next of kin, for the publication of any potentially identifiable images or data included in this article.

## Author contributions

HB-F contributed to conception and design of the study. PS-C, LM, PW, and HB-F recruited all patients and filled out the protocols. ED-N and EH-A managed β-endorphin analyses. MG-L, SR-G, and AS-L processed the samples at the biobank. PW organized the database. CL performed the statistical analysis. PW, CL, MB-F, MM-M, and HB-F wrote the first draft of the manuscript, contributed to manuscript revision, read, and approved the submitted version.

## Funding

This study was funded by Alicia Koplowitz Foundation (Research Grant, 2020).

## Conflict of interest

Author HB-F was employed by company Korian. In the last 24 months, HB-F received lecture fees from Shire. He is Principal Investigator (PI) of an iPFIS research contract (www.isciii.es; IFI16/00039) and co-PI of a MINECO research grant (RTI2018-101857- B-I00); recipient of: 1) a FIPSE Grant, and 2) an IDIPHIPSA intensification grant; involved in two clinical trials (MENSIA KOALA, NEWROFEED Study; ESKETSUI2002); CEO of Haglaia Solutions; member of the Advisory Board of ITA Salud Mental. Author Maria Rodrigo-Yanguas is the recipient of an iPFIS research contract (www.isciii.es; IFI16/00039). Author MM-M is the recipient of a CDTI grant (FEDER funded; IDI-20180701, file 00107278). The remaining authors declare that the research was conducted in the absence of any commercial or financial relationships that could be construed as a potential conflict of interest.

## Publisher's note

All claims expressed in this article are solely those of the authors and do not necessarily represent those of their affiliated organizations, or those of the publisher, the editors and the reviewers. Any product that may be evaluated in this article, or claim that may be made by its manufacturer, is not guaranteed or endorsed by the publisher.

## Abbreviations

ADHD: attention deficit hyperactivity disorder
BE: beta-endorphins
NSSI: non-suicidal self-injury
SA: suicide attempt
SIB: self-injurious behavior.
